# π-Diradical Aromatic Soot Precursors in Flames

**DOI:** 10.1021/jacs.1c05030

**Published:** 2021-08-02

**Authors:** Jacob W. Martin, Laura Pascazio, Angiras Menon, Jethro Akroyd, Katharina Kaiser, Fabian Schulz, Mario Commodo, Andrea D’Anna, Leo Gross, Markus Kraft

**Affiliations:** † Department of Chemical Engineering and Biotechnology, University of Cambridge, CB3 0AS Cambridge, United Kingdom; ‡ Cambridge Centre for Advanced Research and Education in Singapore, 138602 Singapore; § IBM Research − Zurich, Säumerstrasse 4, 8803 Rüschlikon, Switzerland; ∥ Istituto di Scienze e Tecnologie per l’Energia e la Mobilità Sostenibile, CNR, P.le Tecchio 80, 80125 Napoli, Italy; ⊥ Dipartimento di Ingegneria Chimica, dei Materiali e della Produzione Industriale, Università degli Studi di Napoli, Federico II P.le V. Tecchio, 80, 80125 Napoli, Italy; # Nanyang Technological University, 639798 Singapore

## Abstract

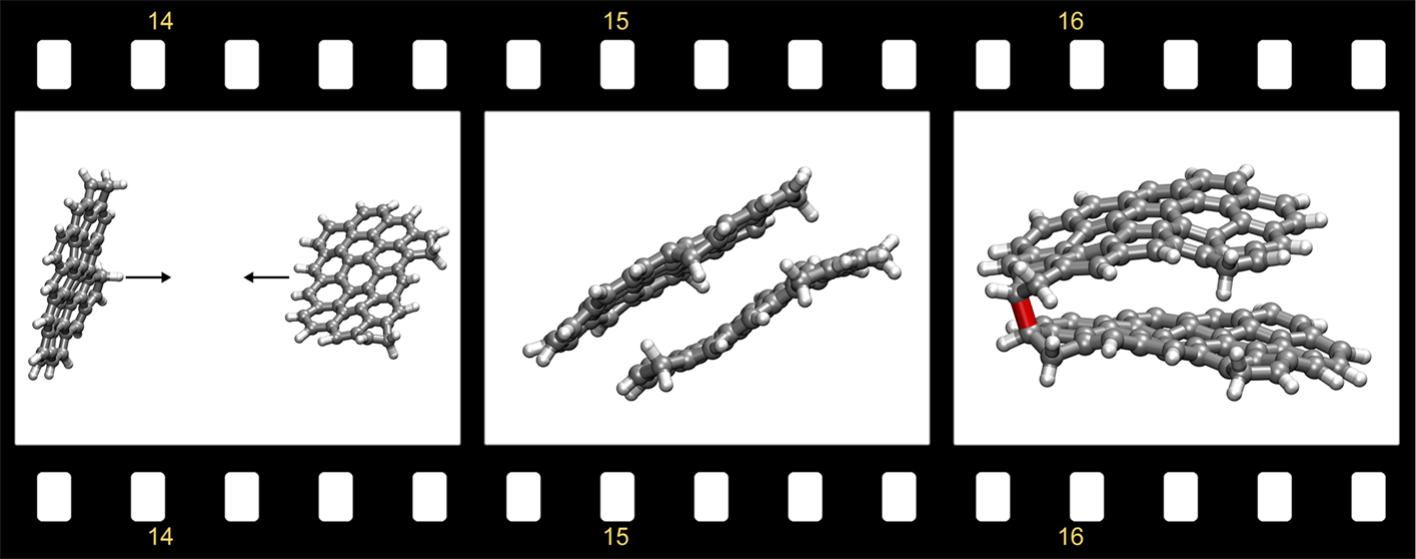

Soot emitted from incomplete combustion of hydrocarbon fuels contributes to global warming and causes human disease. The mechanism by which soot nanoparticles form within hydrocarbon flames is still an unsolved problem in combustion science. Mechanisms proposed to date involving purely chemical growth are limited by slow reaction rates, whereas mechanisms relying on solely physical interactions between molecules are limited by weak intermolecular interactions that are unstable at flame temperatures. Here, we show evidence for a reactive π-diradical aromatic soot precursor imaged using non-contact atomic force microscopy. Localization of π-electrons on non-hexagonal rings was found to allow for Kekulé aromatic soot precursors to possess a triplet diradical ground state. Barrierless chain reactions are shown between these reactive sites, which provide thermally stable aromatic rim-linked hydrocarbons under flame conditions. Quantum molecular dynamics simulations demonstrate physical condensation of aromatics that survive for tens of picoseconds. Bound internal rotors then enable the reactive sites to find each other and become chemically cross-linked before dissociation. These species provide a rapid, thermally stable chain reaction toward soot nanoparticle formation and could provide molecular targets for limiting the emission of these toxic combustion products.

## Introduction

Soot emitted into the atmosphere contributes to global warming, and when deposited on ice, soot lowers ice’s albedo, increasing melting.^1^ Recent estimates place the atmospheric radiative forcing of black carbon at 0.5–1.0 W/m^2^, similar to that of methane.^1^ Additionally, soot and other <2.5 μm combustion products (PM_2.5_) have been directly correlated with increased morbidity and respiratory diseases.^2^ Most pressing is preliminary evidence that an increase of only 1 μm/m^3^ of PM_2.5_ in the urban environment is associated with an 11% increase in COVID-19 related deaths (in preliminary data from the USA).^3^ Recent lockdown measures also demonstrated how quickly soot emissions can drop in the atmosphere with a 12% reduction in PM_2.5_ emissions in 50 major cities around the world, indicating that air quality can rapidly recover.^4^ Flame synthesized carbonaceous nanoparticles could also be functionalized to produce useful quantum nanodots for sensing and bioimaging.^5^ Carbonaceous aerosols are also not limited to earth but litter our universe as interstellar dust and are found in the atmospheres of planets and moons, such as Titan.^6^

**Figure 1:**
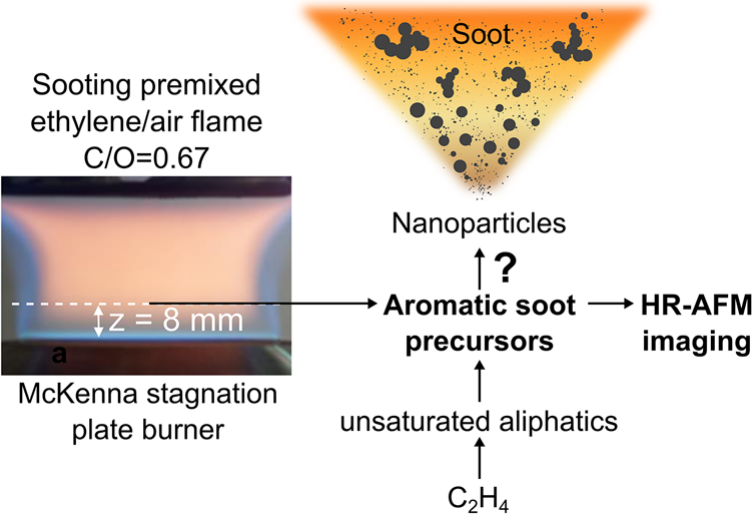
Aromatic soot precursor collection: Burner configuration, collection location, and schematic of soot formation.

No predictive model yet exists for carbonaceous nanoparticle formation, inhibiting our ability to eliminate these pollutants from combustion systems^7^ as well as our ability to produce and tune new nanomaterials. The critical transformation is inception (or nucleation) where gas phase aromatic soot precursors cluster to form nanoparticles (see Figure 1). Three main requirements have been found for this transformation: (1) The species involved must sustain a chain reaction where reactivity or condensability is maintained through subsequent monomer additions.^8,9^ (2) The molecules must be thermally stable with bond energies or intermolecular energies in excess of approximately −167 kJ/mol required for thermal stability at temperatures in the flame where soot begins to form (>1500 K).^10,11^ (3) High collision efficiencies are required to explain the rapid formation of nanoparticles in flames.^9,12,13^ To date, no soot precursor has been found that is able to achieve these three requirements.

Electron paramagnetic resonance spectroscopy demonstrates that π-radicals are dominant in early soot nanoparticles and appear to be critical in their inception.^14^ π-electrons are delocalized in aromatic species that contain six-membered rings and are therefore unreactive.^15^ Localization of π-radicals has long been suggested to provide increased reactivity.^10,16−19^ The past decade has seen significant work on aromatics with extended zigzag edges, such as acenes, which have been shown to localize π-electrons and cross-link.^10,17−19^ However, few of these extended zigzag edges are seen in flame aromatics^20,21^ and the cross-links are not expected to be thermally stable in a flame.^22^ Mass spectroscopic evidence recently demonstrated the existence of five-membered ring π-radicals in soot-forming regions of the flame.^9,23^ Recent images of the aromatic molecules in flames have also revealed these partially saturated pentagonal edges on aromatic molecules.^20,21^ Using electronic structure theory, we demonstrated that these partially saturated edges form highly localized π-radicals,^12,22,24^ which were initially hypothesized by Abrahamson in 1977.^16^ Spectroscopic measurements of these radicals have been undertaken in the astrochemistry community, with their fluorescence suggested to be involved in the unidentified emission from the Red Rectangle proto-planetary nebula.^25^ Of interest for formation mechanisms, localization of π-electrons was found to allow for multiple reactive sites on a single aromatic molecule (with the simplest being a diradical),^26^ which is a requirement for sustaining a chain reaction.

In this work, an aromatic soot precursor imaged with high resolution atomic force microscopy is found to possess a triplet diradical ground state. Localization of π-electrons on non-hexagonal rings is found to be required for this Kekulé aromatic to form the high-spin ground state. These species allow for barrierless reactions with thermally stable bonds capable of a chain reaction. Finally, quantum molecular dynamics simulations reveal rapid reactions between diradical soot precursors, enabled by internal rotors.

## Results and Discussion

**Figure 2:**
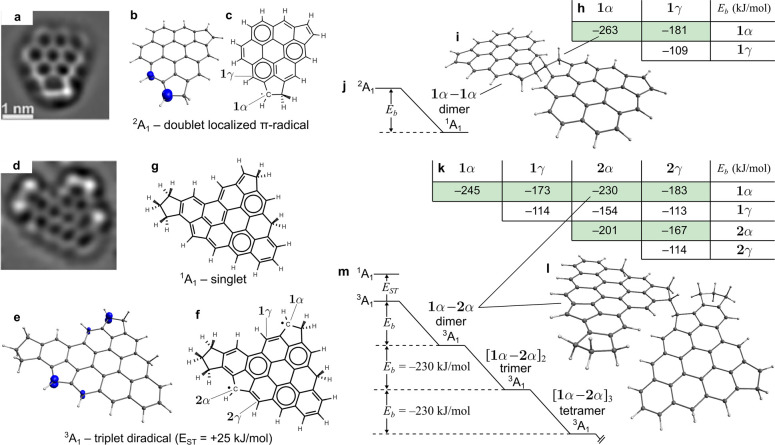
Imaging, electronic structure, and reactions of aromatic radicals. (a, d) HR-AFM images of aromatic soot precursor species (Laplace-filtered sharing the same scale shown in (a)). (b, e) Spin density surfaces (iso = 0.025 au) for the electronic ground state. (c, f, g) The dominant Kekulé structures are also shown. (h, k) The bond energy *E*_b_ in kJ/mol is shown for each cross-link. (i, l) The geometries of one such cross-link. (j, m) Reaction mechanisms for the doublet monoradical and the triplet diradical with the later allowing subsequent polymerization with no loss in reactivity.

Non-contact atomic force microscopy (nc-AFM) revealed a variety of aromatic soot precursors with localized electronic states. Figure 2a shows a coronene structural motif with two rim-based pentagonal rings, one of which is partially saturated, named **1** in this paper (the partial saturation has been confirmed with further imaging and negative ion resonance imaging; see Supporting Information). We have recently demonstrated that a significant concentration of these sites is expected in the flame as hydrogen is added and abstracted from the pentagonal rings’ edges (i.e., being in partial equilibrium with H^•^/H_2_).^26^Figure 2b shows the electron spin density determined from electronic structure calculations revealing the presence of a doublet localized π-radical. This is seen from their spin density which does not decrease with increasing size, showing pinned electronic edge states (a variety of other non-hexagonal rings and methylene type species are also shown to be localized in the Supporting Information). Therefore, while these states are resonantly stabilized, with spin density shared among multiple aromatic carbons, they are localized to the edge, maintaining their spin density and therefore high reactivity with enlargement.

Imaging with nc-AFM also suggests that the molecule shown in Figure 2d) is present in the flame. This molecule features a partially protonated rim-based pentagonal ring and embedded pentagonal ring, labeled **1** and **2**, respectively, in Figure 2f (the structural assignments are discussed in detail in the Supporting Information). Of significance to soot formation, this species was computed to possesses a ground state that is a triplet, *E*_ST_ > 0, with closed-shell and open-shell singlet states lying 25 and 55 kJ/mol above the triplet ground state, respectively. The energy gap between the closed-shell singlet and open-shell triplet states, *E*_ST_, provides a reliable metric for diradical character,^27^ where *E*_ST_ → 0 suggests an increasing diradical character (diradicaloid) and *E* > 0 indicates a true diradical with a triplet ground state. Some well-known diradicaloids, those that contain diradical character, were also found in the flame, including pentacene (protonated in the imaging) and *p*-quinodimethane (see Supporting Information). It has been found for *p*-quinodimethane that thermal excitation of this species into its triplet state allows for a chain polymerization reaction to proceed in the condensed phase.^27,28^ However, this species is the first evidence of a species that is a true diradical with high-spin ground state. Figure 2e shows the electron density and Kekulé of this state with spin density concentrated at the rim in a similar configuration to the doublet.

This diradical aromatic is uncommon in that it is a high-spin Kekulé aromatic. A Kekulé aromatic possesses a chemical structure where each aromatic carbon has a valence of 3 from a combination of single and double bonds; see Figure 2g. Kekulé aromatic species composed of six-membered rings have only been found to possess a singlet ground state and can only attain a triplet ground state for a non-Kekulé configuration of rings (e.g., the recently synthesized heptauthrene^29^ and the extended triangulenes^30,31^). The stabilization of high-spin ground states in Kekulé aromatics has recently been demonstrated in synthesized nanographene structures with two fluorene-like moieties.^32^ These high-spin ground states in Kekulé aromatics demonstrate that truly localized states can exist due to topological defects, i.e., non-hexagonal rings.^27^ (Further details concerning the role of aromaticity in stabilizing the high-spin ground state can also be found in the Supporting Information.)

The reactivity and thermal stability of cross-links formed between the localized π-radicals shown in Figure 2d are computed with electronic structure theory. Figure 2 shows the computed bond energies for the possible cross-links between sites **1** and **2** denoting the partially saturated rim-based pentagonal ring-type (e.g., acenapythenyl) and fluorenyl-type edges, respectively, where the α and γ carbon atoms are the most spin-rich and reactive (see Figure 2c,f). The unstacked configurations were chosen to provide the best comparison between site reactivities without interference from the dispersion interactions in the overlapping geometries.^12^ As mentioned, it has been found experimentally that bonds lower than approximately 167 kJ/mol in magnitude are rapidly degraded in hydrocarbon flames.^10,12^ This cutoff provides various possible configurations of interest (labeled in green in Figure 2). The strongest bonds formed were between **1**α–**1**α sites (with the geometry most closely related to 1,1′,2,2′-tetrahydro-1,1′-biacenaphthylene), **2**α–**2**α (with a geometry most closely related to 9,9′-bifluorene), and **1**α–**2**α with approximately half the bond strength of the C–C single bond in ethane but double that of a bond between two π-radicals on six-membered rings.^15^ Reactions between the γ sites were not found to be thermally stable in the flame but may be important after soot has condensed. Minimal differences are found between the bond energy between the **1**α site for the mono- and diradicals (≈5%), or with the smaller acenapythenyl dimer we have previously calculated^12^ (≈1.5%), showing that reactivity is not dependent on neighboring radicals nor on the size of the aromatic strongly suggesting edge localization. Figure 2b shows the polymerization of the **1**α–**2**α sites. Critically, after two triplet π-diradicals (^3^A_a_) form a dimer involving their α sites, the triplet state endures, allowing for further barrierless reactions to occur. We confirmed that, for the **1**α–**2**α reactions, the bond energy for the dimer + monomer → trimer and, subsequently, the tetramer provides the same bond energy of 230 kJ/mol (we also confirmed the bonds are thermodynamically favored at flame temperatures and that a pairing of both reactive sites is unlikely; see Supporting Information). These reactions therefore can proceed in a chain reaction with no reduction in reactivity, which we call polymerization of aromatic rim-linked hydrocarbons (PARLH). The PARLH mechanism is similar to the clustering of hydrocarbons by the radical-chain reactions (CHRCR) mechanism recently proposed by Johnansson et al. in that the critical species are the π-radical,^9^ but differ in that the reactions are entirely between localized π-radicals and the chain reaction is sustained with π-diradicals.

**Figure 3:**
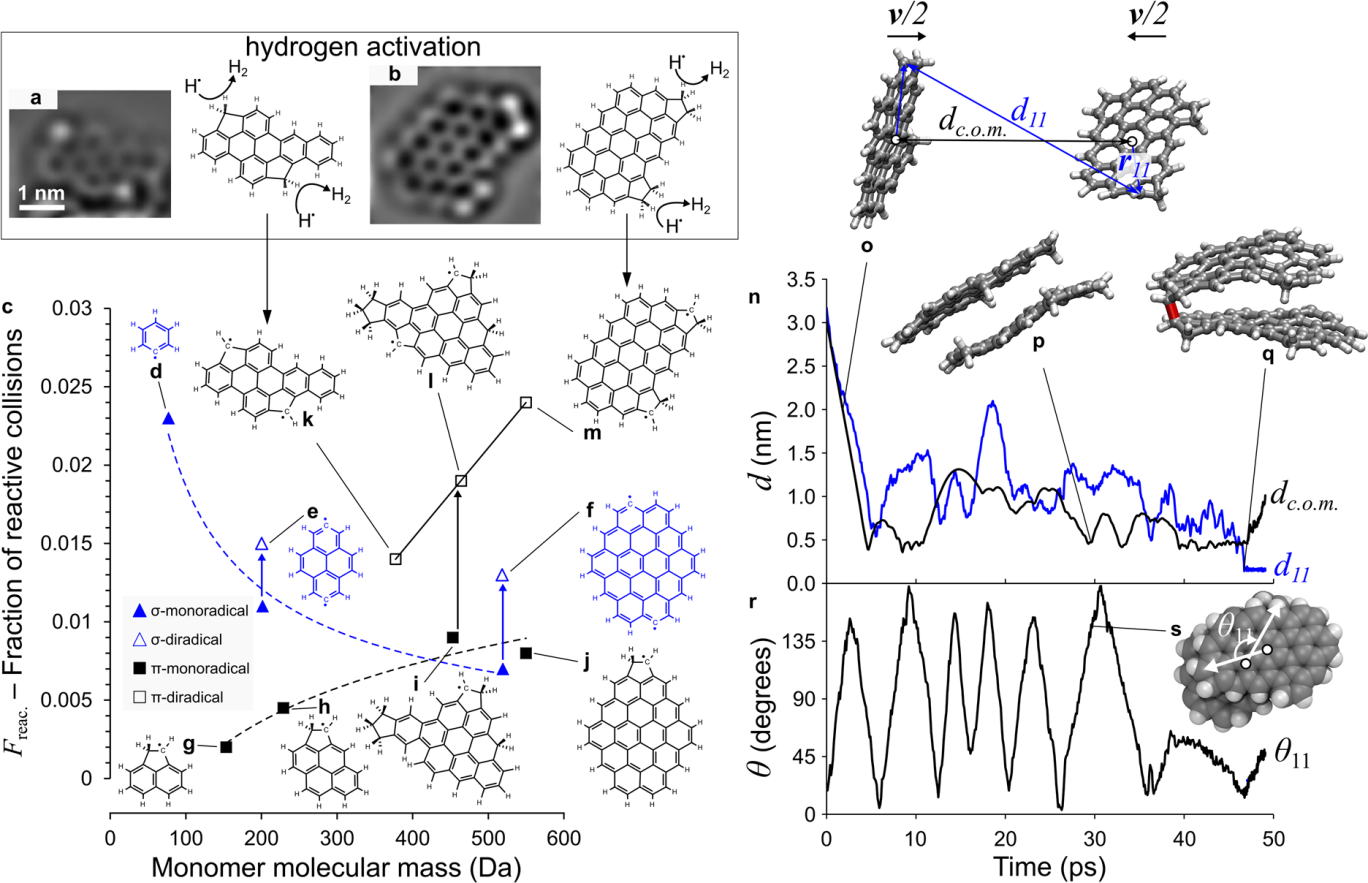
Radical recombination of aromatic radicals. (a, b) Formation of diradicals from hydrogen abstraction of species imaged with nc-AFM from Commodo et al.^21^ (c) Fraction of effective reactive collisions determined using QM/MM simulations for (d–f) σ-monoradicals and σ-diradicals with filled and open triangle symbols, respectively. (g–j) π-Monoradicals filled square symbols. (k–m) π-Diradicals open square symbols derived from HR-AFM structures.^21^ (n) A single reactive trajectory is shown for the diradical in (m). (o) Distances between the centers of mass (*d*_C.O.M._) and the reactive sites that bond, *d*_11_, are plotted. (o–q) Insets show the geometries of the (o) species approaching, (p) stacking unbonded, and (q) stacking and bonding. (r) Angle, θ_11_, between the vectors, ***r***_11_, from the center of mass to the reactive sites (see o) shows the rotation preceding the bond formation. (s) Inset shows a different orientation of the (p) geometry with the direction of rotation in the plane of the aromatic highlighted.

Quantum molecular dynamics is used to study the reaction dynamics for a variety of recombining localized π-radicals and σ-radicals found in the flame. Figure 3a,b shows some π-diradicals that were theoretically prepared from hydrogen abstraction (a common reaction in the flame^26^) of species observed in nc-AFM^21^ (the triplet ground states are demonstrated for these species in the Supporting Information). The σ-radicals are involved in the extension of the aromatic domains with acetylene^13^ and have been suggested to be involved in many soot inception mechanisms.^8,13,33^Figure 3c shows the fraction of effective collisions, *F*_reac._, where a bond is formed during the collision and provides a means of suggesting possible trends in the collision efficiency (see Supporting Information for implementation and comparison with radical recombination rates and Movie 1 for a representative collision between the diradical in Figure 2e). As σ-radicals enlarge (Figure 3d–f), their *F*_reac._ reduces, which has been previously demonstrated with reactive molecular dynamics.^34^ σ-Diradicals are found to provide a modest enhancement in the reactivity over the monoradical. For π-radicals (Figure 3g–j), the opposite trend is found with increasing molecular enlargement: *F*_reac._ increases, significantly so for π-diradicals (Figure 3k–m).

To explore the reason for this enhancement, the largest π- and σ-diradical species, shown in Figure 3m,f, respectively, are compared. Movie 2 shows a representative trajectory for the σ-diradical, where the bond forms upon initial collision. Movie 3 shows a σ-diradical forming during dissociation, which happens less frequently. Both Movies 2 and 3 show the bond formed when the two molecules are not stacked but when the aromatic planes are close to collinear. In fact, trajectories were found where the molecules were stacked for an extended time with the reactive sites frequency approaching each other; however, no bonds were formed when the aromatics were stacked (see Movie 4). This follows from the orthogonality constraints on the sp^2^ hybridized σ-radicals where the orbitals maximally overlap when the carbon atoms are collinear with the aromatic planes.

The dynamics are significantly more complex between two π-diradicals, and therefore, we considered a larger species (see Movie 5) and computed some time series metrics to aid with the analysis. Figure 3n,o shows the distances between the centers of mass for the fragments as well as between the reactive sites. The distance between centers of mass indicates that the aromatic planes are stacking and physically interacting before the bond is formed (Figure 3p). Significant modulation in the distance between the reactive sites is seen before the bond is formed at 41.5 ps and the length is constrained (Figure 3q). To further explore this, the angle between the reactive sites is computed in Figure 3r,s. The reactive sites are found to follow a sinusoidal pattern, demonstrating a stably bound internal rotor is present. In this trajectory, internal rotors allow the reactive sites to find each other and bond before the molecules dissociate. Figure S10 compares the dissociation times for this species with the large σ-diradical. They are found to possess similar dissociation lifetimes due to many collisions leading to a physically bound dimer. We can then consider the fraction of collisions where the reactive sites approach each other, *F*_appr._ (*r*_cutoff_ < 0.3 nm). For the σ-diradical, this fraction is almost double that of the π-diradical, most likely due to the steric issues associated with the saturated carbon site in the latter case. However, the fraction of reactive collisions, *F*_reac._, resulting in a bond forming the π-diradical is approximately double that of the σ-diradical, suggesting the increased lifetime of the dimer enhances the reactions only between π-radicals due to a physically bound internal rotor. Figure S11 shows that this trend is seen for all species where the dissociation time increases with molecular size, while only for the π-radicals is a positive correlation found between the fraction of effective collisions and the dissociation lifetime. It should also be mentioned that reactive collisions were only considered between the most reactive α sites and, therefore, these simulations underestimate and only capture the most significant reactions. More work is required to compute the collision efficiency using more cost-effective methods. Electrostatic effects may also enhance the clustering if polar curved aromatics possess these reactive sites, particularly for interactions with charged chemi-ions which also need to be explored.^35^

These quantum molecular dynamics simulations provide the first evidence for an enhancement due to internal rotors,^13,36^ which is significant for localized π-radicals and can lead to highly efficient reactions approaching that of species that are known to rapidly recombine in the flame such as phenyl.^37^ Preliminary experimental evidence for such a mechanism has been seen for benzyl recombination.^38^ For this smallest localized π-radical, a rapid reaction rate was found that surpassed the high pressure limit when nearing its vapor point where physical interactions become important. HR-AFM imaging also revealed flat species cross-linked via pentagonal rings that could form through dehydrogenation of a rim-linked species.^20,21,39^ Preliminary mass spectroscopic evidence has also been found for clustering of soot precursor aromatics in the 200–1000 Da range.^40−42^ However, the role of physically stabilized rotationally activated reactions between π-diradicals has yet to be demonstrated experimentally.

To fully assess localized π-diradicals as potential soot precursors, their concentration in the flame must be further explored and a kinetic mechanism developed. In order to develop a kinetic mechanism, the impact of the molecular size and different localized π-radicals on the pressure and temperature dependent rates needs to be understood in more detail as well as fragmentation and termination pathways. This will require new approaches to describe the rotationally activated reaction mechanism revealed in the QM/MM simulations. Concerning the concentration of such radicals, our preliminary computational study of rim-based pentagons (acenapthlyene-type) in partial equilibrium with H^•^/H_2_ in the flame revealed that diradicals can form in significant concentrations if multiple rim-based pentagonal rings are present.^26^ Similar analysis is required for the variety of localized π-radical types highlighted in this paper. Another interesting observation is that, due to the resonance stability of edge localized π-radicals, their reaction with molecular oxygen has been shown to be slow at flame temperatures (e.g., for benzyl^43^), suggesting their concentration could grow as does propargyl (known to be the primary route to benzene^37^) for the same reason. Atomic oxygen, which can be added via ozone addition, has been found to react readily with fluorenyl-type localized π-radicals^44^ and could provide a potential route to eliminating these sites in flames. Preliminary evidence suggests significant reduction in soot formation with ozone injection.^45^ These types of bonds could also help explain the strongly bonded and stacked aromatic species found to be critical for the optical response of carbon nanodots.^46^ Finally, such PARLH (or CHRCR^9^ involving also σ-radicals) chain reactions could provide insights into the rapid formation of particulates on planets/moons such as Titan and carbonaceous interstellar dust, where simulations suggest such aromatics with high collision efficiency are required.^6^

## Conclusions

In conclusion, we have shown evidence for a triplet π-diradical that is able to fulfill many of the requirements for carbonaceous nanoparticle formation by providing a chain reaction, bonding strongly enough for stability at flame temperatures and reacting rapidly through physically stabilized internal rotors. Given significant concentrations of these species can be demonstrated in the flame, the PARLH mechanism proposed could provide a feasible pathway to soot formation. These results also provide evidence for a pathway involving both chemical and physical interactions of aromatic radicals, long sought in combustion chemistry^9,13,16,47−50^ and most clearly articulated, perhaps, in 1989 by Harris and Weiner: “Once coagulated they will quickly become chemically knit together since a significant fraction of the aromatic species are radicals”.^47^

## Experimental Details

### Non-contact Atomic Force Microscopy

Incipient nanoparticles were produced using an atmospheric-pressure laminar premixed ethylene–air flame stabilized on a McKenna burner having a diameter of 6 cm (see Figure 1). The cold gas velocity was set at 9.8 cm/s with a carbon to oxygen (C/O) atomic ratio fixed at 0.67. Incipient soot particles were collected from the flame by means of a high-dilution horizontal tubular probe positioned at a distance from the burner surface of 8 mm. At such a flame location, the particle size distribution, measured by differential mobility analysis, is unimodal with a maximum number density for particles with a size of about 2.5 nm; this location is at the onset of soot formation.^20^ Following similar earlier investigations, combustion products were sampled through a small orifice, *i.e.*, 200 μm, located on the bottom side of the stainless-steel probe (1 cm outer diameter and a thickness of 0.05 cm) and mixed with N_2_, to rapidly provide a dilution ratio of 1:(3·10^3^). This sampling procedure prevents particles from coagulating and allows quenching of the chemical reactions throughout the sampling line. Flame temperature profiles, with and without the probe, have been reported previously.^21^ For particle/aromatics collection, a stainless-steel aerosol filter holder containing a quartz filter (Whatman QMA grade, 47 mm) was positioned on-line downstream of the dilution tubular probe. Gas temperature at the filter location was 350 K. At this temperature and with the high dilution condition, the condensation of gas-phase PAHs should be disfavored up to the size of ovalene.

We used a home-built STM/AFM setup operating in ultra-high vacuum and at low temperature (*T* = 5 K). Flash heating was used to deposit the aromatic soot precursors onto the substrate. A qPlus force sensor^51^ was operated in frequency modulation mode with a carbon monoxide functionalized tip.^52^ The AFM images are recorded at constant height, at *V* = 0 V bias voltage. The resonance frequency was *f*_0_ = 28.8 kHz, the quality factor *Q* ∼ 100 000, and we used an oscillation amplitude of *A* = 50 pm. To facilitate structure assignment, several AFM images at different tip–molecule distances where acquired for a given molecule and, if accessible, also images of the frontier orbitals densities by STM (see Supporting Information). Comparison between the species found in AFM with Raman analysis and mass spectrometry confirms they are representative of the species found in the gas phase.^20,23^

### Electronic Structure Calculations

The collection of molecules was geometry optimized using the hybrid density functional theory B3LYP/6-311G(d,p). Molecular energies were calculated using the hybrid meta-GGA DFT method M06-2X/cc-pVTZ in the software Gaussian 16.^53^ This level of theory has been found to provide molecular energies to within 10 kJ/mol of experiments^54^ and accurately reproduced bond energies to within 8 kJ/mol to experimentally derived values and reaction rates between aromatic radicals to within an order of magnitude.^26^ Singlet-triplet energy was calculated using the geometries from B3LYP/6-311G(d,p) and the DLPNO-CCSD(T)/cc-pVTZ method in the software ORCA,^55^ which is known to accurately reproduce energies and singlet-triplet gaps to within 3 kJ/mol at this level of theory.^56^ The T1 diagnostic was found to be below 0.2 in all cases, indicating a single spin configuration is appropriate.

### Molecular Dynamics

A dynamic study on the dimerization of π-radicals under flame conditions is performed using mixed classical molecular mechanics (MM), also known as molecular dynamics, simulations, and quantum mechanics/molecular mechanics (QM/MM) simulations. A series of homobinary collisions between the π-radicals are performed with classical MM simulations. Before studying the binary collisions, structural equilibrium of PAH radicals was performed in the canonical ensemble (NVT) at temperatures of 1500 K. Constant temperature is maintained by a chain of Nosé–Hoover thermostats with a damping constant of 200 fs. Afterward, binary collisions between PAH radicals are performed in the canonical ensemble (NVT) with a time step of 1 fs. To achieve statistical significance, a total of 1000 binary collisions are performed for each case. The initial relative center of mass distance between the two colliding PAH molecules/radicals was 30 Å, which is larger than the effective intermolecular interaction distance between PAHs. The relative translational velocities are set equal to the average speed in the Maxwell–Boltzmann distribution at 1500 K. The orientation of the starting molecules is randomly sampled in three axes. The classical MM simulations are performed using GROMACS. The intramolecular interactions are described using the OPLS-AA force field. The dispersion interactions are described by the isoPAHAP force field based on benchmark SAPT(DFT) calculations.^11^ This force field has been successfully applied to investigate the clustering of aromatic species and accurately reproduces the virial coefficient of benzene. The distance between two reactive sites is then tracked during the whole simulation. When the two reactive sites are at a distance lower than 3 Å, the simulation is switched to QM/MM to determine whether a bond forms between the reactive sites. The QM/MM simulations are performed using GROMACS coupled with ORCA software. The reacting parts of the system are treated quantum mechanically, with the remainder being modeled using the force field (see Supporting Information). The interactions between the QM and MM subsystems are handled within the ONIOM approach. Twelve different monomers have been selected with different sizes and radical types. The quantum mechanical region consists of the pentagonal rings and the adjacent hexagonal rings for the two π-radical site types and the hexagonal ring for the σ-radical (see Supporting Information). The broken symmetry unrestricted method (BS-UM06-2X/def2-SVP) is employed to simulate the QM regions with correct dissociation dynamics.

## Supporting Information Available

Movie 1: QM/MM reactive dimerization of imaged π-diradical (MP4)
Movie 2: QM/MM early reactive dimerization of large σ-diradical (MP4)
Movie 3: QM/MM late reactive dimerization of large σ-diradical (MP4)
Movie 4: QM/MM nonreactive dimerization of large σ-diradical (MP4)
Movie 5: QM/MM reactive dimerization of large π-diradical (MP4)
Further high resolution atomic force microscopy images, localization of π-radicals, quantum mechanics molecular mechanics simulation details, and dimer dissociation lifetimes (PDF)

## References

[ref1] Bond T. C.; et al. Bounding the role of black carbon in the climate system: A scientific assessment. Journal of Geophysical Research Atmospheres 2013, 118, 5380–5552. 10.1002/jgrd.50171.

[ref2] Landrigan P. J.; Fuller R.; Acosta N. J.; Adeyi O.; Arnold R.; Baldé A. B.; Bertollini R.; Bose-O’Reilly S.; Boufford J. I.; Breysse P. N.; et al. The Lancet Commission on pollution and health. Lancet 2018, 391, 462–512. 10.1016/S0140-6736(17)32345-0. 29056410

[ref3] Wu X.; Nethery R.; Sabath M.; Braun D.; Dominici F. Air pollution and COVID-19 mortality in the United States: Strengths and limitations of an ecological regression analysis. Science advances 2020, 6, eabd4049 10.1126/sciadv.abd4049. 33148655 PMC7673673

[ref4] Rodríguez-Urrego D.; Rodríguez-Urrego L. Air quality during the COVID-19: PM2. 5 analysis in the 50 most polluted capital cities in the world. Environ. Pollut. 2020, 266, 115042 10.1016/j.envpol.2020.115042. 32650158 PMC7333997

[ref5] Russo C.; Apicella B.; Ciajolo A. Blue and green luminescent carbon nanodots from controllable fuel-rich flame reactors. Sci. Rep. 2019, 9, 14566 10.1038/s41598-019-50919-1. 31601923 PMC6787054

[ref6] Lavvas P.; Sander M.; Kraft M.; Imanaka H. Surface chemistry and particle shape: processes for the evolution of aerosols in Titan’s atmosphere. Astrophys. J. 2011, 728, 80 10.1088/0004-637X/728/2/80.

[ref7] Kittelson D.; Kraft M. Particle formation and models. Encyclopedia of Automotive Engineering 2014, 1–23. 10.1002/9781118354179.auto161.

[ref8] D’Anna A.; Violi A.; D’Alessio A.; Sarofim A. F. A reaction pathway for nanoparticle formation in rich premixed flames. Combust. Flame 2001, 127, 1995–2003. 10.1016/S0010-2180(01)00303-0.

[ref9] Johansson K.; Head-Gordon M.; Schrader P.; Wilson K.; Michelsen H. Resonance-stabilized hydrocarbon-radical chain reactions may explain soot inception and growth. Science 2018, 361, 997–1000. 10.1126/science.aat3417. 30190399

[ref10] Wang H. Formation of nascent soot and other condensed-phase materials in flames. Proc. Combust. Inst. 2011, 33, 41–67. 10.1016/j.proci.2010.09.009.

[ref11] Totton T. S.; Misquitta A. J.; Kraft M. A quantitative study of the clustering of polycyclic aromatic hydrocarbons at high temperatures. Phys. Chem. Chem. Phys. 2012, 14, 4081–94. 10.1039/c2cp23008a. 22337251

[ref12] Martin J. W.; Hou D.; Menon A.; Pascazio L.; Akroyd J.; You X.; Kraft M. Reactivity of Polycyclic Aromatic Hydrocarbon Soot Precursors: Implications of Localized π-Radicals on Rim-Based Pentagonal Rings. J. Phys. Chem. C 2019, 123, 26673–26682. 10.1021/acs.jpcc.9b07558.

[ref13] Frenklach M.; Mebel A. M. On the mechanism of soot nucleation. Phys. Chem. Chem. Phys. 2020, 22, 5314–5331. 10.1039/D0CP00116C. 32096528

[ref14] Vitiello G.; De Falco G.; Picca F.; Commodo M.; D’Errico G.; Minutolo P.; D’Anna A. Role of radicals in carbon clustering and soot inception: A combined EPR and Raman spectroscopic study. Combust. Flame 2019, 205, 286–294. 10.1016/j.combustflame.2019.04.028.

[ref15] Mou Z.; Uchida K.; Kubo T.; Kertesz M. Evidence of σ-and π-Dimerization in a Series of Phenalenyls. J. Am. Chem. Soc. 2014, 136, 18009–18022. 10.1021/ja509243p. 25394519

[ref16] Abrahamson J. Saturated platelets are new intermediates in hydrocarbon pyrolysis and carbon formation. Nature 1977, 266, 323–327. 10.1038/266323a0.

[ref17] Small D.; Rosokha S. V.; Kochi J. K.; Head-Gordon M. Characterizing the Dimerizations of Phenalenyl Radicals by ab Initio Calculations and Spectroscopy: σ-Bond Formation versus Resonance π-Stabilization. J. Phys. Chem. A 2005, 109, 11261–11267. 10.1021/jp054244n. 16331910

[ref18] Koley D.; Arunan E.; Ramakrishnan S. Computational investigations on covalent dimerization/oligomerization of polyacenes: Is it relevant to soot formation?. J. Comput. Chem. 2012, 33, 1762–1772. 10.1002/jcc.23014. 22610914

[ref19] Zhang H. B.; You X.; Wang H.; Law C. K. Dimerization of polycyclic aromatic hydrocarbons in soot nucleation. J. Phys. Chem. A 2014, 118, 1287–1292. 10.1021/jp411806q. 24491159

[ref20] Schulz F.; Commodo M.; Kaiser K.; De Falco G.; Minutolo P.; Meyer G.; D’Anna A.; Gross L. Insights into incipient soot formation by atomic force microscopy. Proc. Combust. Inst. 2019, 37, 885–892. 10.1016/j.proci.2018.06.100.

[ref21] Commodo M.; Kaiser K.; De Falco G.; Minutolo P.; Schulz F.; D’Anna A.; Gross L. On the early stages of soot formation: Molecular structure elucidation by high-resolution atomic force microscopy. Combust. Flame 2019, 205, 154–164. 10.1016/j.combustflame.2019.03.042.

[ref22] Menon A.; Martin J. W.; Akroyd J.; Kraft M. Reactivity of Polycyclic Aromatic Hydrocarbon Soot Precursors: Kinetics and Equilibria. J. Phys. Chem. A 2020, 124, 10040–10052. 10.1021/acs.jpca.0c07811. 33202128

[ref23] Sabbah H.; Commodo M.; Picca F.; De Falco G.; Minutolo P.; D’Anna A.; Joblin C. Molecular content of nascent soot: Family characterization using two-step laser desorption laser ionization mass spectrometry. Proc. Combust. Inst. 2021, 38, 1241–1248. 10.1016/j.proci.2020.09.022. 33850480 PMC7610591

[ref24] Gentile F. S.; Picca F.; De Falco G.; Commodo M.; Minutolo P.; Causà M.; D’Anna A. Soot inception: A DFT study of σ and π dimerization of resonantly stabilized aromatic radicals. Fuel 2020, 279, 118491 10.1016/j.fuel.2020.118491.

[ref25] Pino T.; Féraud G.; Bréchignac P.; Bieske E. J.; Schmidt T. W. Laboratory spectroscopy of PAHs. Proc. Int. Astron. Union 2013, 9, 247–257. 10.1017/S1743921313015950.

[ref26] Menon A.; Martin J. W.; Leon G.; Hou D.; Pascazio L.; You X.; Kraft M. Reactive localized π-radicals on rim-based pentagonal rings: properties and concentration in flames. Proc. Combust. Inst. 2021, 38, 565–573. 10.1016/j.proci.2020.07.042.

[ref27] Stuyver T.; Chen B.; Zeng T.; Geerlings P.; De Proft F.; Hoffmann R. Do Diradicals Behave Like Radicals?. Chem. Rev. 2019, 119, 11291–11351. 10.1021/acs.chemrev.9b00260. 31593450

[ref28] Issaris A.; Vanderzande D.; Gelan J. Polymerization of a p-quinodimethane derivative to a precursor of poly (p-phenylene vinylene)—Indications for a free radical mechanism. Polymer 1997, 38, 2571–2574. 10.1016/S0032-3861(96)00468-5.

[ref29] Su X.; Li C.; Du Q.; Tao K.; Wang S.; Yu P. Atomically Precise Synthesis and Characterization of Heptauthrene with Triplet Ground State. Nano Lett. 2020, 20, 6859–6864. 10.1021/acs.nanolett.0c02939. 32787160

[ref30] Li J.; Sanz S.; Castro-Esteban J.; Vilas-Varela M.; Friedrich N.; Frederiksen T.; Peña D.; Pascual J. I. Uncovering the triplet ground state of triangular graphene nanoflakes engineered with atomic precision on a metal surface. Phys. Rev. Lett. 2020, 124, 177201 10.1103/PhysRevLett.124.177201. 32412280

[ref31] Mishra S.; Beyer D.; Eimre K.; Kezilebieke S.; Berger R.; Gröning O.; Pignedoli C. A.; Müllen K.; Liljeroth P.; Ruffeux P.; et al. Topological frustration induces unconventional magnetism in a nanographene. Nat. Nanotechnol. 2020, 15, 22–28. 10.1038/s41565-019-0577-9. 31819244

[ref32] Lombardi F.; Myers W. K.; Ma J.; Liu J.; Feng X.; Bogani L. Dynamical nuclear decoupling of electron spins in molecular graphenoid radicals and biradicals. Phys. Rev. B: Condens. Matter Mater. Phys. 2020, 101, 094406 10.1103/PhysRevB.101.094406.

[ref33] Michelsen H. A. Probing soot formation, chemical and physical evolution, and oxidation: A review of in situ diagnostic techniques and needs. Proc. Combust. Inst. 2017, 36, 717–735. 10.1016/j.proci.2016.08.027.

[ref34] Mao Q.; Hou D.; Luo K. H.; You X. Dimerization of Polycyclic Aromatic Hydrocarbon Molecules and Radicals under Flame Conditions. J. Phys. Chem. A 2018, 122, 8701–8708. 10.1021/acs.jpca.8b07102. 30351104

[ref35] Martin J. W.; Botero M.; Slavchov R. I.; Bowal K.; Akroyd J.; Mosbach S.; Kraft M. Flexoelectricity and the formation of carbon nanoparticles in flames. J. Phys. Chem. C 2018, 122, 22210–22215. 10.1021/acs.jpcc.8b08264.

[ref36] Schuetz C. A.; Frenklach M. Nucleation of soot: Molecular dynamics simulations of pyrene dimerization. Proc. Combust. Inst. 2002, 29, 2307–2314. 10.1016/S1540-7489(02)80281-4.

[ref37] Hansen N.; Cool T. A.; Westmoreland P. R.; Kohse-Höinghaus K. Recent contributions of flame-sampling molecular-beam mass spectrometry to a fundamental understanding of combustion chemistry. Prog. Energy Combust. Sci. 2009, 35, 168–191. 10.1016/j.pecs.2008.10.001.

[ref38] Luther K.; Oum K.; Sekiguchi K.; Troe J. Recombination of benzyl radicals: dependence on the bath gas, temperature, and pressure. Phys. Chem. Chem. Phys. 2004, 6, 4133–4141. 10.1039/b407074g.

[ref39] Pascazio L.; Martin J. W.; Menon A.; Hou D.; You X.; Kraft M. Aromatic penta-linked hydrocarbons in soot nanoparticle formation. Proc. Combust. Inst. 2021, 38, 1525–1532. 10.1016/j.proci.2020.09.029.

[ref40] Happold J.; Grotheer H.-H.; Aigner M. In Combustion Generated Fine Carbonaceous Particles; Bockhorn H., D’Anna A., Sarofim A., Wang H., Eds.; KIT Scientific Publishing: Karlsruhe, Germany, 2009; Chapter 18, pp 277–288.

[ref41] Carbone F.; Canagaratna M. R.; Lambe A. T.; Jayne J. T.; Worsnop D. R.; Gomez A. Exploratory analysis of a sooting premixed flame via on-line high resolution (APi-TOF) mass spectrometry. Proc. Combust. Inst. 2019, 37, 919–926. 10.1016/j.proci.2018.08.020.

[ref42] Faccinetto A.; Irimiea C.; Minutolo P.; Commodo M.; D’Anna A.; Nuns N.; Carpentier Y.; Pirim C.; Desgroux P.; Focsa C.; Mercier X. Evidence on the formation of dimers of polycyclic aromatic hydrocarbons in a laminar diffusion flame. Commun. Chem. 2020, 3, 112 10.1038/s42004-020-00357-2. PMC9814144 36703341

[ref43] Pelucchi M.; Cavallotti C.; Faravelli T.; Klippenstein S. H-Abstraction reactions by OH, HO2, O, O2 and benzyl radical addition to O2 and their implications for kinetic modelling of toluene oxidation. Phys. Chem. Chem. Phys. 2018, 20, 10607–10627. 10.1039/C7CP07779C. 29387837

[ref44] Frenklach M.; Liu Z.; Singh R. I.; Galimova G. R.; Azyazov V. N.; Mebel A. M. Detailed, sterically-resolved modeling of soot oxidation: Role of O atoms, interplay with particle nanostructure, and emergence of inner particle burning. Combust. Flame 2018, 188, 284–306. 10.1016/j.combustflame.2017.10.012.

[ref45] Sun W.; Gao X.; Wu B.; Ombrello T. The effect of ozone addition on combustion: Kinetics and dynamics. Prog. Energy Combust. Sci. 2019, 73, 1–25. 10.1016/j.pecs.2019.02.002.

[ref46] Liu C.; Singh A. V.; Saggese C.; Tang Q.; Chen D.; Wan K.; Vinciguerra M.; Commodo M.; De Falco G.; Minutolo P.; et al. Flame-formed carbon nanoparticles exhibit quantum dot behaviors. Proc. Natl. Acad. Sci. U. S. A. 2019, 116, 12692–12697. 10.1073/pnas.1900205116. 31182580 PMC6601003

[ref47] Harris S. J.; Weiner A. M. A picture of soot particle inception. Symp. (Int.) Combust., [Proc.] 1989, 22, 333–342. 10.1016/S0082-0784(89)80039-6.

[ref48] Miller J. H. The kinetics of polynuclear aromatic hydrocarbon agglomeration in flames. Symp. (Int.) Combust., [Proc.] 1991, 23, 91–98. 10.1016/S0082-0784(06)80246-8.

[ref49] Frenklach M.; Wang H. Detailed modeling of soot particle nucleation and growth. Symp. (Int.) Combust., [Proc.] 1991, 23, 1559–1566. 10.1016/S0082-0784(06)80426-1.

[ref50] Kholghy M. R.; Kelesidis G. A.; Pratsinis S. E. Reactive polycyclic aromatic hydrocarbon dimerization drives soot nucleation. Phys. Chem. Chem. Phys. 2018, 20, 10926–10938. 10.1039/C7CP07803J. 29542752

[ref51] Giessibl F. J. High-speed force sensor for force microscopy and profilometry utilizing a quartz tuning fork. Appl. Phys. Lett. 1998, 73, 3956–3958. 10.1063/1.122948.

[ref52] Gross L.; Mohn F.; Moll N.; Liljeroth P.; Meyer G. The chemical structure of a molecule resolved by atomic force microscopy. Science 2009, 325, 1110–1114. 10.1126/science.1176210. 19713523

[ref53] Frisch M. J.; Gaussian 16, Rev. B.01; Gaussian, Inc.: Wallingford, CT, 2016.

[ref54] Zhao Y.; Truhlar D. G. The M06 suite of density functionals for main group thermochemistry, thermochemical kinetics, noncovalent interactions, excited states, and transition elements: two new functionals and systematic testing of four M06-class functionals and 12 other functionals. Theor. Chem. Acc. 2008, 120, 215–241. 10.1007/s00214-007-0310-x.

[ref55] Neese F. The ORCA program system. Wiley Interdiscip. Rev.: Comput. Mol. Sci. 2012, 2, 73–78. 10.1002/wcms.81.

[ref56] Ghafarian Shirazi R.; Neese F.; Pantazis D. A. Accurate Spin-State Energetics for Aryl Carbenes. J. Chem. Theory Comput. 2018, 14, 4733–4746. 10.1021/acs.jctc.8b00587. 30110157

