# Supplementary material for: π-Diradical Aromatic Soot Precursors in Flames
Source: J Am Chem Soc. 2021 Aug 2;143(31):12212–9. doi: 10.1021/jacs.1c05030 (PMC8361428; doi:10.1021/jacs.1c05030)
Supplement: Supplementary file 6 — ja1c05030_si_006.pdf [file ja1c05030_si_006.pdf]

Supplementary Information:
π-Diradical aromatic soot precursors in flames

Jacob W. Martin, Laura Pascazio, Angiras Menon, Jethro Akroyd, and Markus Kraft*

Department of Chemical Engineering and Biotechnology,

Univeristy of Cambridge,

Philippa Fawcett Dr,

Cambridge CB30AS, UK

and

Cambridge Centre for Advanced Research and Education in Singapore,

1 Create Way #05-05 CREATE Tower, Singapore 138602

Mario Commodo

Istituto di Scienze e Tecnologie per l'Energia e la Mobilità Sostenibile,

CNR, P.le Tecchio 80, 80125 Napoli, Italy

Andrea D'Anna

Dipartimento di Ingegneria Chimica,

dei Materiali e della Produzione Industriale Università degli Studi

di Napoli Federico II P.le V. Tecchio, 80, 80125 Napoli, Italy

Katharina Kaiser, Fabian Schulz, and Leo Gross

IBM Research – Zurich,

Säumerstrasse 4, 8803 Rüschlikon, Switzerland

(Dated: June 22, 2021)

I. HIGH RESOLUTION ATOMIC FORCE MICROSCOPY

Extended discussions on the imaging approach can be found in our previous publications and in the main text [1, 2]. We conducted additional analysis on the structural elucidation of the species discussed in the main text. In particular, the differentiation of CH and CH_2 moieties at the edges of the molecules at five-membered rings is challenging and is analyzed and discussed here in detail.

Figure S1 shows extended scanning tunnelling microscopy (STM), density functional theory (DFT) and atomic force microscopy (AFM) data on the first species, labelled IS1 in previous work [2], which was imaged on bilayer NaCl on Cu(111) with a CO functionalized tip. With constant current STM at an applied sample bias $V = 0.2$ V, which is within the transport gap of the molecule, electrons cannot be attached to the molecule and the image contrast is explained by a lowering of the tunnelling barrier for the path through the

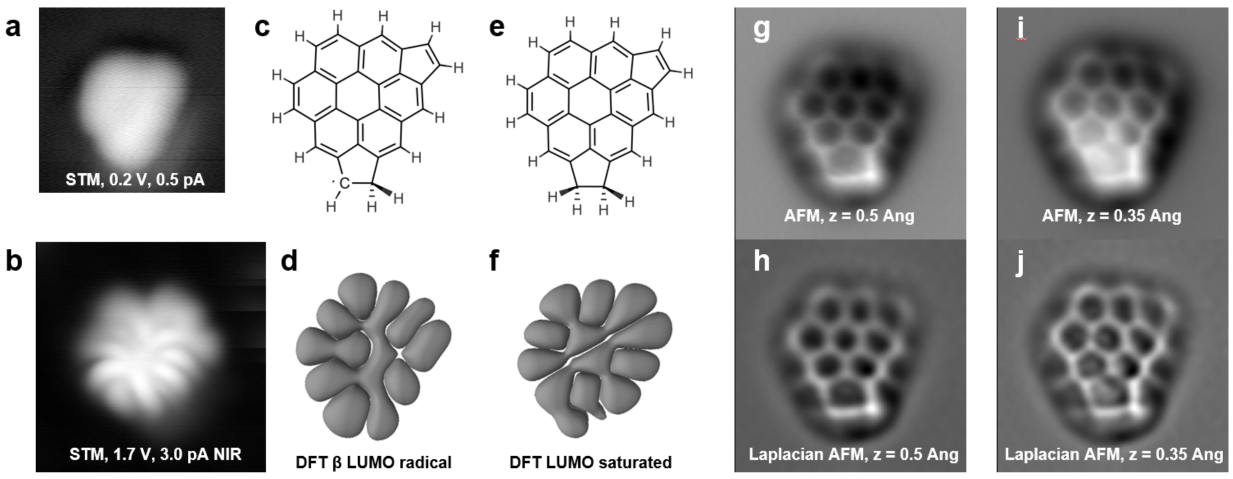

FIG. S1. Additional STM, DFT and AFM data for IS1. STM images at (a) $V = 0.2$ V, $I = 0.5$ pA and (b) $V = 1.7$ V, $I = 3.0$ pA on bilayer NaCl on Cu(111) using a CO functionalised tip. Structure (c) and calculated LUMO density at an iso-surface value of 0.002 of IS1 (d). Structure of a molecule featuring a second CH_2 moiety at the five-membered ring of IS1, resulting in a closed shell structure (e) and its calculated LUMO density (f). Constant height AFM data at different tip height z is shown in (g) and (i), together with the corresponding Laplace filtered images (h) and (j), respectively. A decrease in z corresponds to a decrease in tip-sample distance.

* mk306@cam.ac.uk; Also at Nanyang Technological University, Singapore

molecule [3], revealing little intramolecular contrast, see Fig. S1a.

With STM at $V = +1.7$ V, see Fig. S1b, we could access the negative ion resonance (NIR) of this molecule. At this bias, we attach from the tip, electrons to the first unoccupied state above the Fermi level, *i.e.*, the lowest unoccupied molecular orbital (LUMO). The constant current STM image at resonance can be interpreted as a contour map of the corresponding orbital density [3]. In addition, the usage of a CO functionalized tip gives rise to a partial p-wave character of the tip, with sensitivity to the lateral gradient of the orbital density [4].

We calculated the LUMO at the M06-2X/cc-pVTZ level of theory for the previously assigned molecular structure IS1 [1, 2]. The structure of the molecule is shown in Fig. S1c and the calculated LUMO orbital density (in this case, as the molecule is a radical, corresponding to a singly unoccupied molecular orbital) at an iso-surface value of 0.002 a.u. is shown in Fig. S1d. In Addition, we also calculated the LUMO for a closed shell molecule that features a second CH_2 moiety at the five-membered ring, see its structure shown in Fig. S1e and its calculated LUMO iso-surface in Fig. S1f.

The calculated LUMO density of the radical IS1 (Fig. S1d) fits very well the STM measurement (Fig. S1b), whereas the LUMO of the closed shell structure molecule with an additional CH_2 moiety (Fig. S1f) does not. This electronic characterization by STM and its comparison with the calculated orbital densities firmly corroborates our structural assignment of IS1 of the previous publication [2].

This molecule showcases the challenge in assigning that specific moiety, *i.e.*, the level of saturation of the five-membered ring with a CH_2 moiety. At the position of the CH group in that five-membered ring, the AFM contrast is significantly brighter compared to the contrast on the other CH groups within the molecule (see Fig. S1g and Fig. S1i). This increase in brightness could be explained by a non-planar adsorption geometry, and increased adsorption height at the CH group at the five-membered ring, caused by the neighboring CH_2 moiety, leading to a locally increased adsorption height and tilted adsorption plane of the molecule [5].

Figure S2 shows additional AFM and STM data that was measured on molecule IS56 on bilayer NaCl on Cu(111). Unfortunately, we did not acquire STM images at ionic resonance for that molecule, because the molecule was picked up by the tip before orbital density images were obtained. AFM images of the molecule are shown for different tip heights in Fig. S2. The aliphatic six-membered ring is assigned in comparison with ref. [6] with

high confidence. The CH group at a five-membered ring labelled i) and the CH_2 group at a six-membered ring labelled ii) in Fig. S2 are assigned with high confidence, too. More challenging and of less confidence is the assignment of the saturation of the five-membered ring labelled iii) in Fig. S2. Comparison to molecule IS1, that features a very similar AFM contrast, indicates that this is likely a partially saturated five-membered ring, *i.e.*, with one CH and one CH_2 moiety, as in IS1, suggesting the initially proposed structure IS56 [2]. However, we cannot firmly exclude a five-membered ring with two CH_2 groups, as we are missing STM orbital density images to confirm this challenging structural assignment for this molecule, in contrast to IS1. In the context of this work it is important to note that both possible assignments for this molecule, *i.e.*, IS56 and the hydrogenated radical of IS56 with two CH_2 groups at the five-membered ring, could be interconverted by exchange of atomic hydrogen in the flame [2]. Thus, both possible structural assignments suggest that both molecule IS56 and the hydrogenated radical of IS56 are present in the flame.

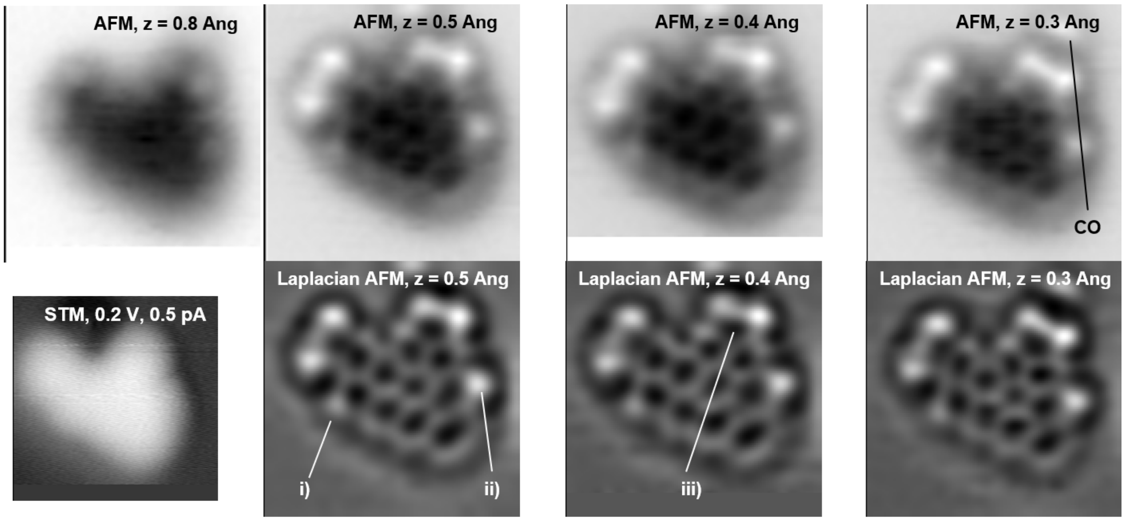

FIG. S2. Additional STM and AFM data for IS56. Constant current STM in gap image at $V = 0.2$ V, $I = 0.5$ pA on bilayer NaCl on Cu(111) using a CO functionalised tip, labelled STM. Constant height AFM data at different tip height z together with the corresponding Laplace filtered images. A decrease in z corresponds to a decrease in tip-sample distance.

II. LOCALISATION OF Π-RADICALS

The localisation of π-radicals can be demonstrated from the spin density for a variety of aromatic radicals with a doublet ground state. Figure S3 shows the isosurface of the spin density calculated for a variety of motifs as a function of molecular size. The spin density is correlated with the resulting reactivity of the carbon atom [7]. We have previously demonstrated the distinction between the delocalised π-radicals (phenalenyl-type) and localised π-radicals for the partially saturated rim pentagon-type (Part. sat. rim pent-type) [8], however, based on the saturated edges found via non-contact AFM [2] other localised π-radicals could be generated from hydrogen addition/abstraction. For benzyl-type radicals these sites have also been referred to as α-radicals due to the concentration of spin density on a primary α-carbon atom [9]. They arise from aromatics that contain non-hexagonal rings, methylene groups or zig-zag edges.

A more concrete definition of localisation has been proposed recently based on aromatic diradicals [7]. The singlet-triplet energy, E_{ST}, is described as a measure of the diradical character. A variety of imaged molecules were found to have diradical character, diradicaloids, with $E_{\mathrm{ST}} < 0$ and $E_{\mathrm{ST}} \to 0$ (see Fig. S4). For species for which a triplet ground state is found, where $E_{\mathrm{ST}} > 0$, these are considered to be localisable states [7]. For aro-

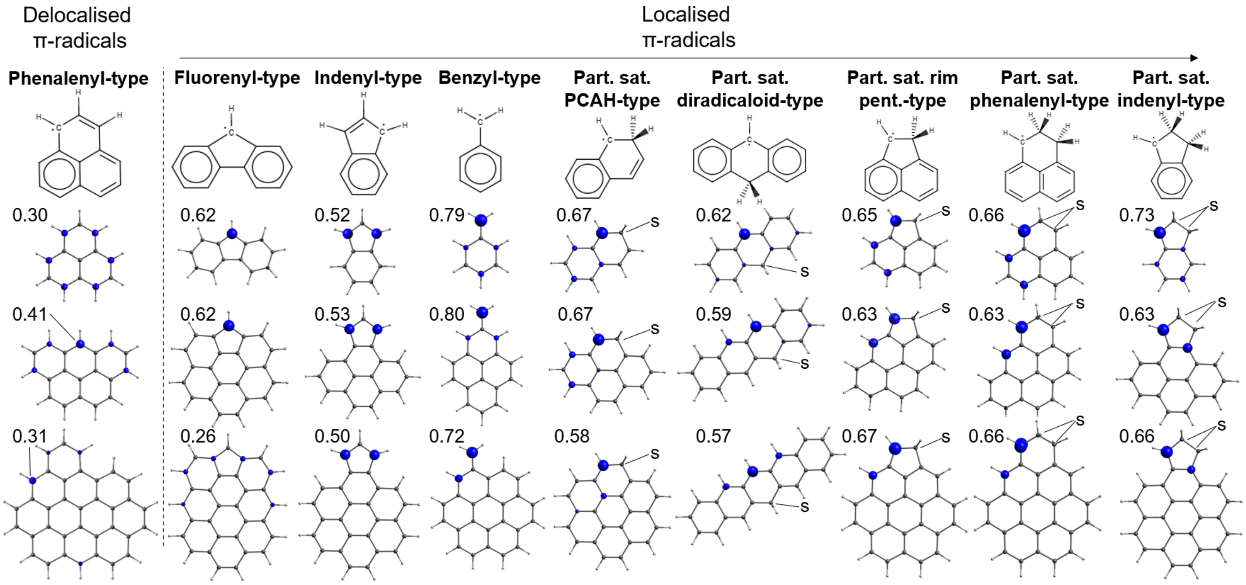

FIG. S3. Spin density (iso=0.025) study of delocalised versus localised π-radicals as a function of molecular size. The Mullikan spin is also shown for the most spin-rich carbon atom.

matic molecules only containing hexagonal ring networks this has only been achieved for non-Kekulé aromatics, however, the species imaged in the main text are all Kekulé aromatics as can be seen from Fig. S4. The stabilisation of the high spin state in these aromatics provides more evidence that these reactive sites as electronically localised and is related to their aromaticity [7].

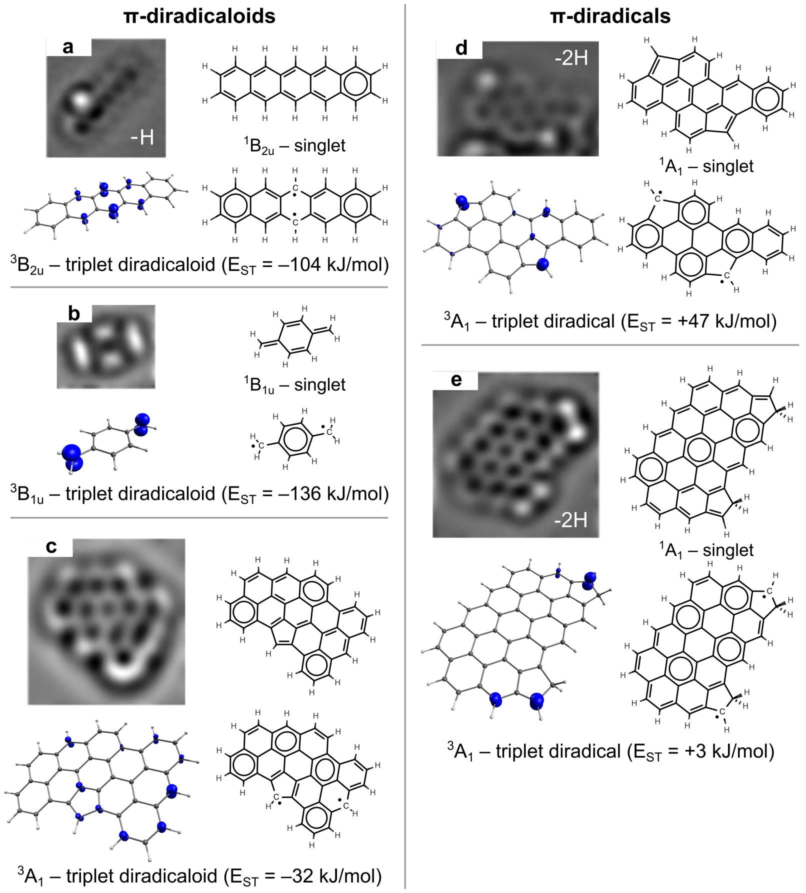

FIG. S4. nc-AFM imaging and electronic structure analysis of π-diradicaloids and π-diradicals. For species a), d) and e) hydrogen was removed from the imaged molecule to provide the diradical(oid) species. Singlet-Triplet energy gaps, E_{ST} are provided to determine diradical character at the DLPNO-CCSD(T)/cc-pVTZ//B3LYP/6-311G(d,p) level of theory.

The Clar construction provides an analysis of the degree of aromatic stabilisation. Sextets (six-membered rings can contain 6 π-electrons that are in resonance) are drawn with a circle and indicate resonantly stabilised 6-member cycles, with more sextets indicating greater stability [10]. Figure S4 reveals that while closed shell Kekulé structures can be drawn for many of these molecules, the open-shell structure often contains more Clar sextets indicating

greater aromatic stability is involved. We have previously used such an analysis to argue for the localisation of the π-radical in the doublet state [11] but it appears a similar effect enables diradical states to be stabilised on aromatic species.

Another indication of localisation is the sustained bond strength as a polymer is constructed. Figure S5a-c shows the dimer, trimer and tetramer geometries for the $\mathbf{1}\alpha$–$\mathbf{2}\alpha$ series. The binding energy for the addition of one monomer were all found to be –230 kJ/mol for the dimer, trimer and tetramer showing no loss of reactivity. This indicates that the reactivity of one reactive site does not depend on the bonding state of the other reactive site showing the localisation of the reactivity.

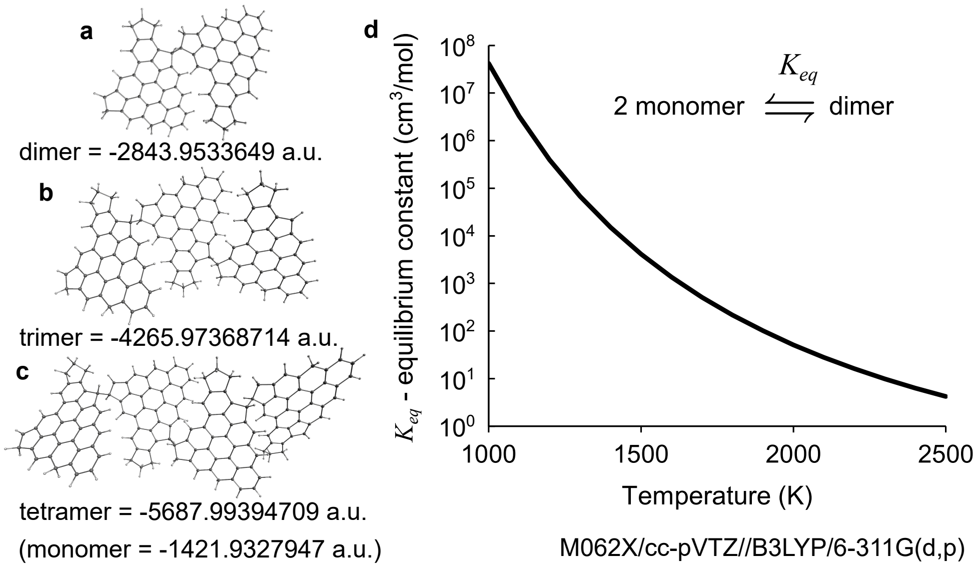

FIG. S5. a-c) Geometries and energies for the dimer, trimer and tetramer. d) Equilibrium constant for dimerisation is computed for the $\mathbf{1}\alpha$–$\mathbf{2}\alpha$ dimer showing that the product is favoured at temperatures found in the flame.

A question arises as to whether these crosslinks are thermally stable from a thermodynamics point of view. Figure S5d shows the equilibrium analysis for the dimerisation of two monomers of $\mathbf{1}\alpha$–$\mathbf{2}\alpha$. Full vibrational analysis of the monomers and dimers as well as the energetics were computed, allowing for the temperature dependent equilibrium constant for dimerisation to be computed, K_{eq}. We find values $K_{eq} > 1$ over the entire range of temperatures seen in hydrocarbon flames (<2500 K). This suggests that once the crosslinks are formed they will be persistent and thermally stable. This result is consistent with our

previous calculations on smaller localised π-radical species [12]. However, little can be said about the forward reaction as the van der Waals interactions were deliberately ignored by preparing the structure in an unstacked configuration and a reduced equilibrium constant would be a potentially more useful approach [13].

Finally, the possibility of a π-diradical pair where both reactive sites crosslink is explored. Such a reaction would terminate a chain reaction driven by π-diradicals. Figure S6 shows the geometry optimized structure of the $\mathbf{1}\alpha$–$\mathbf{2}\alpha\times 2$ reaction. From this optimized geometry a potential energy scan between one of the crosslinks was performed to determine the energy difference between having one and two crosslinks between the diradical species (using the broken symmetry unrestricted BS-UM06-2X/cc-pVTZ level of theory). The energy difference was found to be –81 kJ/mol. This is significantly lower bond energy compared with unstrained single crosslink between these sites of –230 kJ/mol. This can be explained due to the strained geometry of the paired diradical where bond lengths are 1.60Åcompared with the single crosslink of 1.58 Å. This bond energy for the paired diradical is not thermally stable in the flame. It should also be mentioned that such pairing of diradicals is unlikely between non-identical flame diradicals where the two unreacted localised π-radicals would need to be accessible via rotation relative to the initial crosslink.

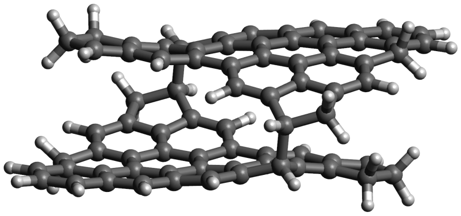

FIG. S6. Paired π-diradicals reacted through $\mathbf{1}\alpha$–$\mathbf{2}\alpha$ bonds optimized at the M06-2X/cc-pVTZ//B3LYP/6-311G(d,p) level of theory.

III. QUANTUM MECHANICS MOLECULAR MECHANICS SIMULATIONS

In this work, the crosslinking reactions have been simulated using a combination of quantum mechanics (QM) and molecular mechanics (MM). The reactive sites are treated quantum mechanically, with the remainder being modeled using the iso-PAHAP force field for

the intermolecular interactions and OPLS-AA force field for the intramolecular interactions. The QM/MM division connects the systems along chemical bonds (see Fig. S7). At the bonds that connects the QM and MM subsystems, link atoms are introduced (labelled LA in Fig. S7). These link atoms are present as a hydrogen atom in the QM calculation step and are not physically present in the MM subsystem, but the forces on it are computed in the QM step and are distributed over the two atoms in the bond as will be discussed. The QM region is simulated with ORCA software using broken symmetry spin unrestricted SCF calculations (BS-M06-2X/def2-SVP). Molecules with three different types of reactive sites are simulated: a σ-radical and two π-radical site types - partially saturated rim pentagon type (defined as **1**α in Fig. 2g) and fluorenyl type (defined as **2**α in Fig. 2g) - (Fig. S7).

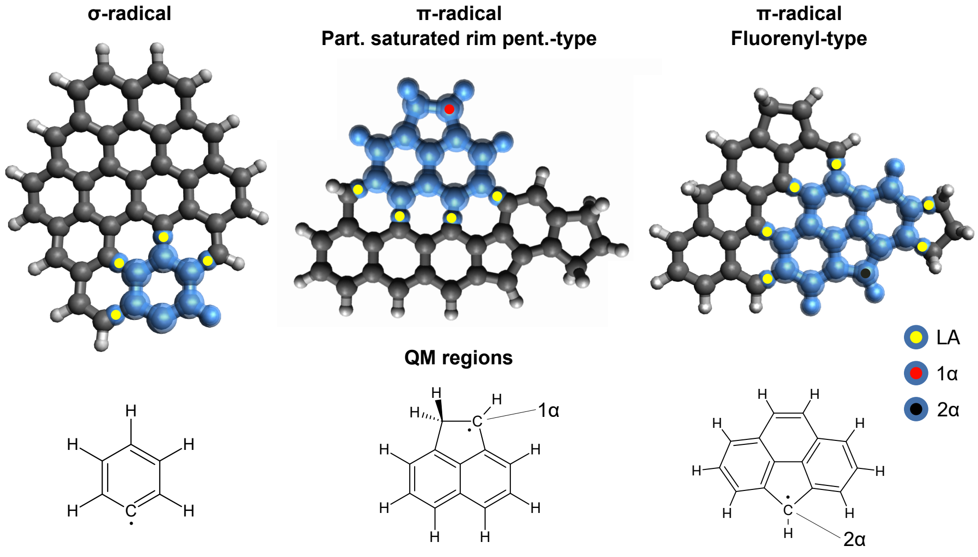

FIG. S7. Division of the system in a QM subsystem (blue) and an MM subsystem (gray) for the three type of radicals investigated in this work - σ-radical, partially saturated rim pentagon type π-radical (defined as **1**α in Fig. 2g) and fluorenyl type π-radical (defined as **2**α in Fig. 2g). Link atoms (LA) are introduced at the QM/MM boundary to cap the QM subsystem. The link atoms are present as a hydrogen atom in the QM calculation step.

The interactions between the two subsystems are handled within the ONIOM approach by Morokuma and coworkers [14, 15]. In the ONIOM approach, the energy and gradients are first evaluated for the isolated QM subsystem at the desired level of *ab initio* theory. Subsequently, the energy and gradients of the total system, including the QM region, are computed using the molecular mechanics force field and added to the energy and gradients

calculated for the isolated QM subsystems. Finally, in order to correct for counting the interactions inside the QM region twice, a molecular mechanics calculation is performed on the isolated QM subsystem and the energy and gradients are subtracted. This leads to the following expression for the total QM/MM energy (and gradients likewise):

$$E_{\mathrm{tot}} = E_{\mathrm{I}}^{\mathrm{QM}} + E_{\mathrm{I+II}}^{\mathrm{MM}} - E_{\mathrm{I}}^{\mathrm{MM}}, \tag{1}$$

where the subscripts I and II refers to the QM and MM subsystems, respectively. The superscripts indicate at what level of theory the energies are computed.

The sizes of the QM regions (see Fig. S7) have been selected to ensure an accurate electronic description of the reactive sites. This was firstly assumed looking at the similar values of the crosslink energy in 1,1',2,2'-Tetrahydro-1,1'-biacenaphthylene and 9,9'-Bifluorene [8, 12] and in bigger molecules presenting the same π-radical types, indicating that the QM regions selected are appropriate. The QM/MM method have been further benchmarked below:

- Calculation of crosslink energies: Geometry optimisations using the QM/MM method for the crosslinked molecules and monomers have been performed and crosslink energies have been calculated as the difference between the dimer energy and monomers energies (M06-2X/cc-pVTZ, which we have found to provide bond energies to within 4–8 kJ/mol [16, 17]). Energies of the bond between unstacked configurations of π-radicals (crosslinks between **1**α-**1**α sites, **2**α-**2**α sites and **1**α-**2**α sites as defined in Fig. 2g) were chosen as in the main text and are reported and compared with the electronic structure theory calculations in Fig. S8. We found that QM/MM method provide a similar energy to DFT with $\approx 10\%$ of overbinding for the π-radical cases and $\approx 1.5\%$ for the σ-radical case. The overestimation is mainly due to the lower level of theory used in the QM/MM simualations (M06-2X/def2-SVP) with respect to the electronic structure theory calculations (M06-2X/cc-pVTZ). We could not use the same level of theory due the high computational cost of the QM/MM simulations.

- Calculation of the intermolecular interactions between non-bonded stacked larger species: The dispersion interactions of two different stacked configurations have been calculated using classical MM and QM/MM geometry optimisations (see Fig. S9).

The values are in good agreement indicating that the QM/MM approach is able to treat the dispersion forces as well as the classical MM approach with the accurate isoPAHAP forcefield.

- Collision efficiency of phenyl recombination: The collision efficiency β is defined as the ratio of successful collisions (i.e. collisions which form dimers) to the total number of collisions. It is possible to estimate β from the MM/QM/MM simulations of radical-radical collisions as the fraction of effective collisions. We found 23 effective collisions over 1000 collisions for phenyl radicals at 1500 K, resulting in β equal to 0.023. It is important to mention that we have only considered direct collisions and we have not considered the collisions where the collision velocities are offset (impact factor) that would be required to accurately compute the collision efficiency. It is then more

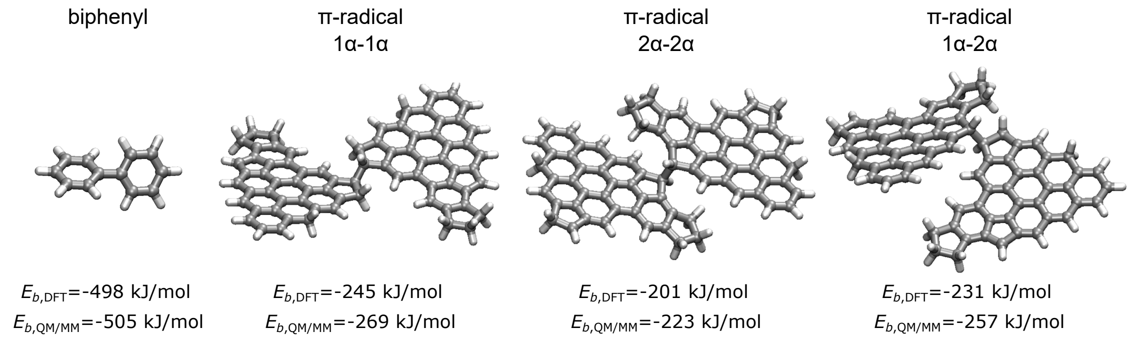

FIG. S8. Energies of the bond between phenyl radicals and between unstacked configurations of π-radicals (crosslinks between 1α-1α sites, 2α-2α sites and 1α-2α sites as defined in Fig. 2g) calculated using electronic structure theory calculations (M06-2X/cc-pVTZ) and QM/MM simualations (M06-2X/def2-SVP).

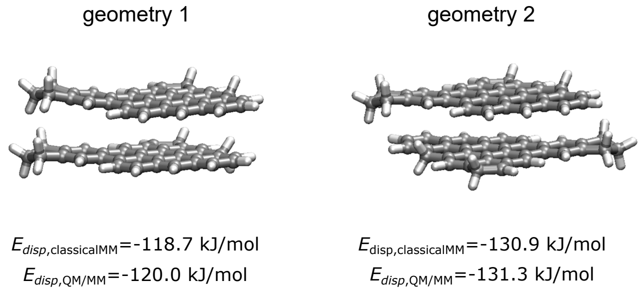

FIG. S9. Dispersion energies between non-bonded stacked larger species calculated using classical MM and QM/MM.

appropriate to refer to it as fraction of effective collisions. However, in case of small molecules, the impact factor for the offset collisions is small, so we do not expect a significant difference between the fraction of effective collisions and the collision efficiency for phenyl radical recombination.

From the collision theory, the dimerization rate (r) of phenyl recombination can be expressed as:

$$r = k \cdot C_{\mathrm{PHE}}^2 = \beta \cdot \sqrt{\frac{4\pi k_b T}{m_{\mathrm{PHE}}}} \cdot d_{\mathrm{PHE}}^2 \cdot C_{\mathrm{PHE}}^2 \tag{2}$$

where k is the rate constant, C_{PHE} is phenyl concentration, β is the collision efficiency, k_b is the Boltzmann constant, T is the temperature, m_{PHE} is the mass of the phenyl radical and d_{PHE} is the collision diameter expressed by:

$$d_{\mathrm{PHE}} = d_{\mathrm{A}} \sqrt{\frac{2 n_{\mathrm{C}}}{3}} \tag{3}$$

where d_A denotes the size of a single aromatic ring and equals to $1.395\sqrt{3}$ Å and n_c is the number of carbon atoms in the PAH monomer. It is then possible to calculate the collision efficiency β from the rate constant k:

$$\beta = \frac{k}{\sqrt{\frac{4\pi k_b T}{m_{\mathrm{PHE}}}} \cdot d_{\mathrm{PHE}}^2} \tag{4}$$

The collision efficiency for phenyl radical combinations ($\beta = 0.020$) derived using $k = 4 \cdot 10^{12}$ cm^3/mol/s taken from the high temperature shock tube study by Jin et al. [18] in Eq. 4 is adopted for comparison. The two values are in reasonable agreement suggesting the results are comparable experimentally measured values for phenyl recombination.

IV. DIMER DISSOCIATION LIFETIMES

Lifetimes for physical dimers were recorded using a distance cut-off of 2 nm between the monomers center of mass to distinguish between monomer and dimer state. The dissociation probability was defined as $N(t)/N(0)$, where $N(t)$ is the number of dimers that survived for at least t picoseconds and $N(0)$ is the initial number of dimers (in similar analysis to that performed by Chakraborty *et al.* [19]). In all the dissociation curves considered herein, there is an initial delay in the $N(t)/N(0)$ plots in which all the dimers survived. To fit the

$N(t)/N(0)$ distributions versus time, we used a mono-exponential function of the following form:

$$\frac{N(t)}{N(0)} = -a \cdot \exp\left(\frac{1}{\tau} \cdot t\right) \tag{5}$$

The initial constant data points are not considered in the fitting. $N(t)/N(0)$ and the exponential fits are shown in Fig. S10.

τ is an indicator of the dimer dissociation lifetimes. As expected, τ increases with molecular mass being dependent on the dispersion force. Figure S11 shows that this trend is seen for all species where the dissociation time increases with molecular size while only for the π-radicals a positive correlation is found between the fraction of effective collisions and the dissociation lifetime.

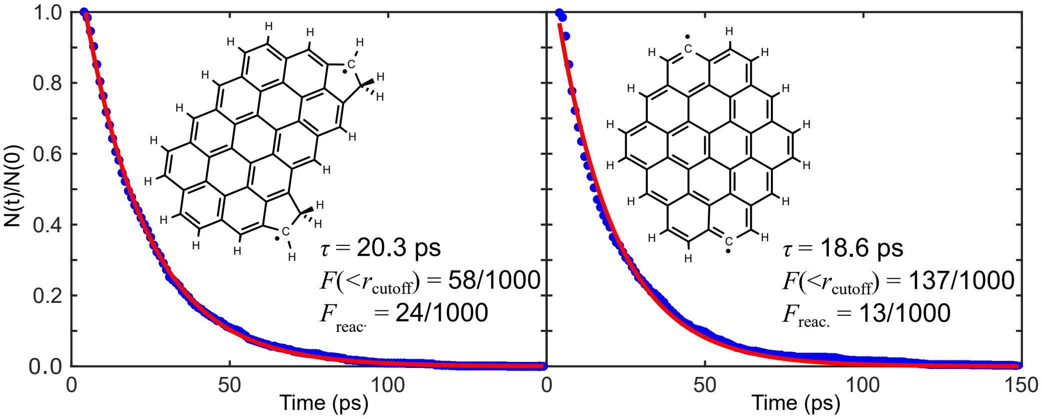

FIG. S10. Plots of $N(t)/N(0)$ and exponential fits for $M_2^* \rightarrow 2M$ dissociation a) M = IS22 and b) M = circumpyrene-2-nyl. The blue points represent the simulation results, whereas the red curves represent the exponential fit (Eq. 5). Inset shows lifetimes, τ, fraction of reactive sites that approached the cutoff, $r_{\text{cutoff}} < 0.3$ Å and the fraction of effective collisions that go on to react, F_{eff}.

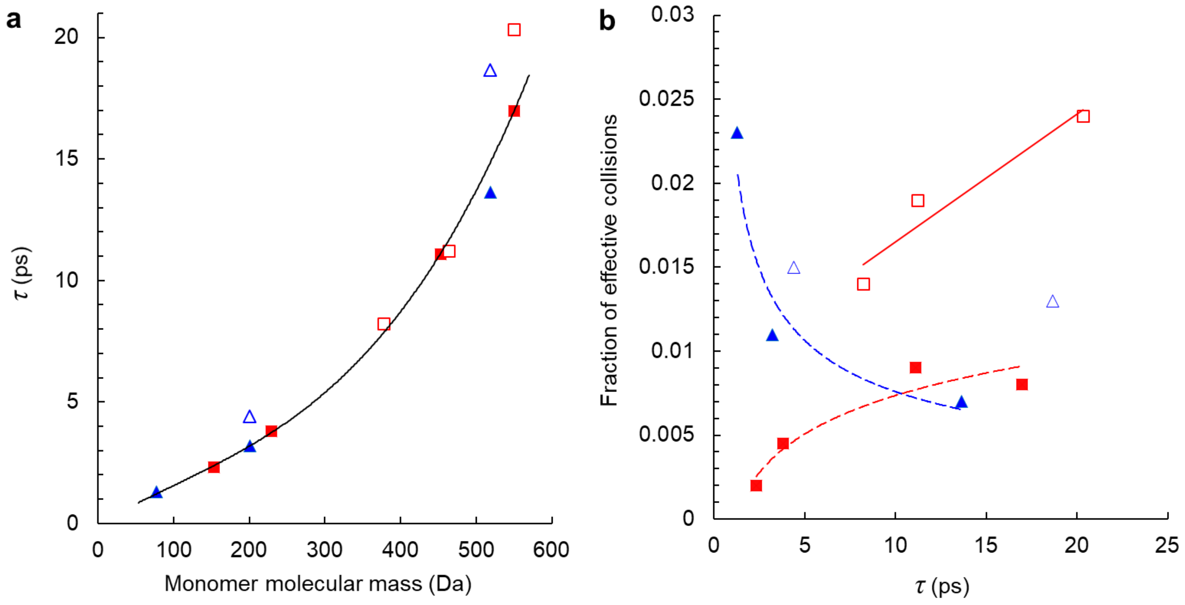

FIG. S11. a) dimer dissociation lifetimes (τ) versus monomer molecular mass and b) fraction of effective collisions versus dimer dissociation lifetimes (τ) for all the species investigated (Fig. 3 d-m): σ-monoradicals (filled triangles), σ-diradicals (open triangles), π-monoradicals (filled squares) and π-diradicals (open squares).

REFERENCES

[1] F. Schulz, M. Commodo, K. Kaiser, G. D. Falco, P. Minutolo, G. Meyer, A. D'Anna, and L. Gross, Insights into incipient soot formation by atomic force microscopy, Proceedings of the Combustion Institute **37**, 885 (2019).

[2] M. Commodo, K. Kaiser, G. De Falco, P. Minutolo, F. Schulz, A. D'Anna, and L. Gross, On the early stages of soot formation: Molecular structure elucidation by high-resolution atomic force microscopy, Combustion and Flame **205**, 154 (2019).

[3] J. Repp, G. Meyer, S. M. Stojković, A. Gourdon, and C. Joachim, Molecules on insulating films: scanning-tunneling microscopy imaging of individual molecular orbitals, Physical Review Letters **94**, 026803 (2005).

[4] L. Gross, N. Moll, F. Mohn, A. Curioni, G. Meyer, F. Hanke, and M. Persson, High-resolution

molecular orbital imaging using a p-wave stm tip, Physical Review Letters **107**, 086101 (2011).

[5] B. Schuler, W. Liu, A. Tkatchenko, N. Moll, G. Meyer, A. Mistry, D. Fox, and L. Gross, Adsorption geometry determination of single molecules by atomic force microscopy, Physical review letters **111**, 106103 (2013).

[6] B. Schuler, Y. Zhang, S. Collazos, S. Fatayer, G. Meyer, D. Pérez, E. Guitián, M. R. Harper, J. D. Kushnerick, D. Pena, *et al.*, Characterizing aliphatic moieties in hydrocarbons with atomic force microscopy, Chemical science **8**, 2315 (2017).

[7] T. Stuyver, B. Chen, T. Zeng, P. Geerlings, F. De Proft, and R. Hoffmann, Do diradicals behave like radicals?, Chemical reviews **119**, 11291 (2019).

[8] J. W. Martin, D. Hou, A. Menon, L. Pascazio, J. Akroyd, X. You, and M. Kraft, Reactivity of polycyclic aromatic hydrocarbon soot precursors: Implications of localized π-radicals on rim-based pentagonal rings, The Journal of Physical Chemistry C **123**, 26673 (2019).

[9] P. Chen, S. Fatayer, B. Schuler, J. N. Metz, L. Gross, N. Yao, and Y. Zhang, The role of methyl groups in the early stage of thermal polymerization of polycyclic aromatic hydrocarbons revealed by molecular imaging, Energy & Fuels , In Press (2021).

[10] M. Solà, Forty years of clar's aromatic π-sextet rule, Frontiers in chemistry **1**, 22 (2013).

[11] A. Menon, J. W. Martin, G. Leon, D. Hou, L. Pascazio, X. You, and M. Kraft, Reactive localized π-radicals on rim-based pentagonal rings: properties and concentration in flames, Proceedings of the Combustion Institute , In Press (2020).

[12] A. Menon, J. W. Martin, J. Akroyd, and M. Kraft, Reactivity of polycyclic aromatic hydrocarbon soot precursors: Kinetics and equilibria, The Journal of Physical Chemistry A 10.1021/acs.jpcc.9b07558 (2020).

[13] M. Frenklach, R. I. Singh, and A. M. Mebel, On the low-temperature limit of haca, Proceedings of the Combustion Institute **37**, 969 (2019).

[14] F. Maseras and K. Morokuma, IMOMM: A new ab initio + molecular mechanics geometry optimization scheme of equilibrium structures and transition states, J. Comp. Chem. **16**, 1170–1179 (1995).

[15] M. Svensson, S. Humbel, R. Froes, T. Matsubara, S. Sieber, and K. Morokuma, ONIOM a multilayered integrated MO + MM method for geometry optimizations and single point energy predictions. a test for Diels-Alder reactions and Pt(P(t-Bu)3)2 + H2 oxidative addition, J. Phys. Chem. **100**, 19357 (1996).

[16] D. Hou and X. You, Reaction kinetics of hydrogen abstraction from polycyclic aromatic hydrocarbons by H atoms, Physical Chemistry Chemical Physics **19**, 30772 (2017).

[17] Y. Zhao and D. G. Truhlar, The m06 suite of density functionals for main group thermochemistry, thermochemical kinetics, noncovalent interactions, excited states, and transition elements: two new functionals and systematic testing of four m06-class functionals and 12 other functionals, Theoretical Chemistry Accounts **120**, 215 (2008).

[18] H. Jin, B. R. Giri, D. Liu, and A. Farooq, A high temperature shock tube study of phenyl recombination reaction using laser absorption spectroscopy, Proc. Combust. Instit. **In Press** (2020).

[19] D. Chakraborty, H. Lischka, and W. L. Hase, Dynamics of pyrene-dimer association and ensuing pyrene-dimer dissociation, The Journal of Physical Chemistry A **124**, 8907 (2020).
